# Advanced Nanoporous and Mesoporous Materials: Emerging Innovations, Multiscale Design, and Future Perspectives

**DOI:** 10.3390/ma19061141

**Published:** 2026-03-15

**Authors:** Monika Fedyna, Jakub Mokrzycki

**Affiliations:** 1 Faculty of Chemistry, Jagiellonian University, ul. Gronostajowa 2, 30-387 Krakow, Poland; 2 Faculty of Energy and Fuels, AGH University of Krakow, al. A. Mickiewicza 30, 30-059 Krakow, Poland; jmokrzycki@agh.edu.pl

## 1. Introduction

Nanoporous and mesoporous materials have become some of the most dynamic and rapidly evolving areas of modern materials science [1]. Their exceptional structural characteristics—including extremely high specific surface areas (significantly exceeding 1000 m^2^ g^−1^, often above 3000 m^2^ g^−1^) [2], precisely controlled pore size distributions, tunable pore connectivity, and chemically versatile surfaces—enable a wide spectrum of functional applications. These materials play a crucial role in adsorption, catalysis, gas storage and separation, environmental remediation, sensing, energy conversion, and drug delivery [3]. Over the past decade, the field has experienced accelerated growth, driven not only by fundamental scientific curiosity but also by urgent global challenges such as climate change, environmental pollution, sustainable energy production, and the growing demand for advanced biomedical technologies.

The ability to tailor porous architectures at multiple length scales—from atomic-level surface chemistry to hierarchical pore networks—positions nanoporous and mesoporous materials at the forefront of next-generation technologies [4]. Importantly, the design of these materials increasingly integrates sustainability principles with performance optimization. As a result, porous materials are no longer viewed solely as high-performance functional solids but as strategic tools in addressing societal and environmental needs [5].

A wide variety of porous materials contribute to this evolving landscape. Biochars and activated carbons, often derived from biomass or agricultural waste, are distinguished by their hierarchical porosity and large surface areas, providing abundant active sites for pollutant adsorption [6,7]. Their relatively low production costs and renewable origin make them particularly attractive for large-scale environmental applications. Metal–organic frameworks (MOFs) and covalent organic frameworks (COFs) offer unprecedented structural tunability, enabling precise control over pore size, topology, and surface functionality [8,9]. These crystalline frameworks allow selective adsorption, catalysis, and molecular encapsulation with molecular-level precision. Zeolites, characterized by their uniform microporous structures and high thermal and chemical stability, function as robust molecular sieves in size- and shape-selective separations [10,11]. Mesoporous silicas, with well-defined and adjustable pore sizes, provide rapid mass transfer and versatile surface functionalization, making them highly adaptable platforms for catalysis and drug delivery [12]. Together, these material classes demonstrate how structural engineering at the nanoscale translates directly into enhanced macroscopic performance.

A defining trend in contemporary porous materials research is the integration of green chemistry and sustainable synthesis strategies. Conventional preparation methods often rely on energy-intensive processes, hazardous solvents, or non-renewable precursors. In contrast, emerging approaches emphasize solvent minimization, the elimination of toxic reagents, and the utilization of renewable or waste-derived feedstocks [13]. Agricultural residues, industrial by-products, and biomass waste streams are increasingly employed as precursors for activated carbons, biochars, and even silica-based materials [14]. These strategies not only reduce environmental impact but also improve economic feasibility and scalability. Sustainability, therefore, is no longer an auxiliary consideration but an intrinsic design parameter in advanced porous material development.

Parallel to experimental advances, computational modeling and data-driven approaches are transforming material discovery and optimization. High-throughput simulations, density functional theory (DFT) calculations, and machine learning algorithms now enable the prediction of structure–property relationships with remarkable accuracy [15]. Instead of relying exclusively on empirical trial-and-error experimentation, researchers increasingly employ predictive frameworks to identify optimal pore architectures, surface functionalities, and compositional parameters. Such integration of computational and experimental methodologies accelerates innovation and facilitates the rational design of application-specific porous systems.

Surface functionalization represents another critical dimension of modern porous material engineering [16]. The incorporation of heteroatoms, metal dopants, specific ligands, or polymeric coatings allows fine-tuning of adsorption selectivity, catalytic activity, hydrophilicity, or biocompatibility. For example, functional groups that selectively interact with CO_2_, SO_x_, or NO_x_ enhance gas capture efficiency, while tailored organic ligands in biomedical systems enable receptor-mediated targeting. In drug delivery, stimuli-responsive polymer coatings can introduce pH-, temperature-, or redox-triggered release mechanisms. Thus, the synergy between pore architecture and surface chemistry defines the operational boundaries of nanoporous and mesoporous materials.

This Special Issue gathers ten original research articles contributed by researchers from different countries and scientific disciplines. The collection reflects the diversity and interdisciplinary nature of porous materials research, spanning biomedical delivery systems, catalytic emission control, greenhouse gas capture, aqueous pollutant removal, microplastic–pollutant interactions, and structure–property relationships in advanced inorganic systems. Together, these studies demonstrate how innovations in synthesis, structural control, and functionalization translate into measurable technological advances.

## 2. Biomedical Applications of Porous Materials

Although porous materials are traditionally associated with catalysis and adsorption, their biomedical relevance has grown substantially.

Mesoporous silica nanoparticles (MSNs) represent a prominent example of porous systems engineered for smart drug delivery (contribution 1). In this study, MSNs were functionalized with biopolymer conjugates, including carboxymethyl chitosan–dopamine and hyaluronic acid–folic acid, enabling active targeting of tumor cells. The intrinsic high surface area and ordered pore network of MSNs provided substantial drug-loading capacity, while polymeric surface modification introduced pH-responsive release behavior. The hybrid nanocarriers demonstrated efficient encapsulation, controlled release in acidic tumor-like environments, and significant reduction in tumor cell viability. Importantly, the work highlights how hierarchical design—combining inorganic mesoporous cores with biologically active surface layers—enables multifunctional therapeutic systems. However, further investigation into biodistribution, long-term toxicity, immune response, and large-scale reproducibility remains essential before clinical translation can be realized.

Biomedical structural applications were explored through the design of gyroid-based Zn–Mg scaffolds with controlled porosity gradients for bone tissue engineering (contribution 2). Unlike conventional homogeneous porous scaffolds, these structures incorporated radial porosity gradients to optimize mechanical stability and biological integration simultaneously. Forward radial gradient designs exhibited superior load-bearing capacity and favorable stress distribution. In vitro and in vivo experiments confirmed good biocompatibility of Zn–2Mg porous scaffolds, demonstrating their potential as biodegradable implants. This study underscores the importance of geometrical complexity and gradient design in achieving a balance between structural integrity and biological performance.

## 3. Gas Pollutant Removal and Catalytic Systems

The mitigation of gaseous pollutants remains a critical environmental priority, and porous materials play an essential role in addressing this challenge.

Activated carbon fibers modified with hopcalite catalysts were investigated for hydrogen chloride (HCl) removal from industrial emissions (contribution 3). Hydrogen chloride is a corrosive and toxic pollutant that poses serious environmental and health risks. The incorporation of hopcalite enhanced the adsorption efficiency to approximately 90%, achieved through synergistic physical adsorption and catalytic chemisorption mechanisms. Importantly, catalyst loading was optimized to maintain the intrinsic microporous structure of activated carbon fibers while increasing the density of reactive sites. This balance between structural preservation and functional enhancement exemplifies rational catalyst–support integration.

Selective catalytic reduction (SCR) of nitrogen oxides (NO_x_) was addressed using titania-based mixed oxide catalysts modified with cerium, iron, or copper (contribution 4). Cerium–titania and iron–titania systems demonstrated high NO conversion across a broad temperature range (200–400 °C) without forming undesirable by-products such as N_2_O. Catalytic performance was closely associated with the presence of Fe^3+^ and Ce^3+^ surface species and the coexistence of Lewis and Brønsted acid sites. The results highlight how redox-active dopants and acid–base properties govern catalytic efficiency and stability.

CO_2_ capture was further explored using Ti-, Zr-, and mixed Ti–Zr oxide-modified montmorillonite clays (contribution 5). Modified clays exhibited enhanced CO_2_ sorption compared to pristine bentonite, attributed to increased microporosity and cation-induced surface interactions. Notably, prolonged exposure to dry CO_2_ induced structural evolution and altered sorption behavior, emphasizing the importance of long-term stability studies in gas capture applications.

## 4. Pollutant Removal from Aqueous Systems

Water purification remains one of the most active research directions in porous materials science.

Activated carbon derived from olive mill solid waste demonstrated high adsorption capacity for methylene blue, achieving up to 95% removal efficiency (contribution 6). The adsorption process exhibited limited sensitivity to pH changes, indicating robustness across diverse aqueous environments. This work illustrates how agricultural waste valorization can provide cost-effective and environmentally sustainable sorbents capable of competing with commercial materials.

The removal of per- and polyfluoroalkyl substances (PFAS) was investigated using granular activated carbon and organoclays (contribution 7). Organoclays showed superior adsorption performance and largely irreversible binding behavior. The study underscores the importance of surface chemistry, hydrophobic interactions, and specific functional groups in capturing persistent and chemically resistant contaminants.

The environmental behavior of 4-methylbenzylidene camphor (4-MBC), a commonly used UV filter, was studied in the context of its adsorption onto textile-derived microplastic fibers (contribution 8). Adsorption experiments conducted in municipal wastewater, Danube River water, and laundry effluent revealed matrix-dependent behaviour, with municipal wastewater promoting the highest adsorption due to elevated organic matter content and ionic strength. The findings demonstrate that microplastic fibers may act as vectors for hydrophobic micropollutants, contributing to their transport and bioavailability in aquatic systems.

## 5. Structure–Property Relationships and Synthetic Control

A crucial aspect of porous material research involves understanding how synthesis parameters influence microstructure and performance.

Nanoporous membranes fabricated from single-crystal nickel-based superalloys (CMSX-4) were studied in relation to microstructural evolution during high-temperature creep (contribution 9). The research demonstrated that mechanical performance depends not only on chemical composition but also on crystallographic alignment, defect distribution, and microstructural homogeneity. Local misorientations created preferential crack propagation pathways under specific loading conditions. These findings emphasize the necessity of precise microstructural control in designing mechanically robust nanoporous membranes.

The synthesis of HKUST-1 metal–organic frameworks using different copper (II) salt precursors was systematically examined (contribution 10). The efficiency of crystallization and final physicochemical properties were strongly dependent on the salt precursor used. Acetate ions promoted rapid nucleation and high yield but could partially incorporate into the framework, generating secondary phases. Chloride ions inhibited crystallization under modulator-free conditions. Furthermore, eliminating mixing during synthesis produced larger, more uniform crystals with reduced porosity. This work highlights the sensitivity of MOF formation to subtle synthetic variables and underscores the importance of parameter optimization in achieving desired structural and functional characteristics. It also brings forward the opportunity of synthesizing MOFs as one of the most broadly applicable groups of porous materials, using relatively environmentally friendly, modulator-free synthesis procedures.

## 6. Future Perspectives

The contributions to this Special Issue collectively highlight several transformative trends that are likely to define the next decade of nanoporous and mesoporous materials research. Looking forward, several strategic directions emerge that integrate scientific insight, technological innovation, and societal impact [17,18,19].

- Sustainability as a Design Imperative

Future porous materials research will increasingly prioritize environmentally responsible synthesis and deployment. Life-cycle analysis, energy-efficient processes, and the use of renewable or waste-derived feedstocks will become standard considerations, rather than optional features. The development of materials that combine high performance with minimal environmental footprint is crucial to meeting global targets in energy sustainability, catalytic processes, water purification, and carbon capture. For example, large-scale production of biochar-based adsorbents from agricultural residues or MOFs synthesized under solvent-minimized conditions, research on efficient and selective catalysts, and low-energy conditions could revolutionize industrial pollutant remediation, while simultaneously addressing circular economy goals.

- Integration of Artificial Intelligence and Computational Materials Discovery

The integration of machine learning, high-throughput simulations, and data-driven optimization is set to transform material design. Predictive models can now anticipate structure–property relationships, enabling rapid identification of optimal pore geometries, surface chemistries, and hybrid material compositions. Future research will increasingly leverage AI-assisted discovery pipelines to accelerate development, minimize experimental iterations, and tailor materials for highly specific applications, from selective gas separation to biomedical targeting. Such approaches may enable the prediction of long-term stability and performance in complex environmental or biological systems, bridging a critical gap between laboratory results and real-world applicability.

- Multiscale and Hierarchical Structural Engineering

The design of hierarchical porosity spanning micro-, meso-, and macropores will continue to emerge as a central paradigm. Such multiscale architectures improve mass transport, increase accessibility to active sites, and enhance mechanical robustness. Computational tools can guide pore network design to optimize kinetics, selectivity, and load-bearing properties. Beyond individual applications, multiscale design may enable multifunctional materials that integrate pollutant capture, catalysis, and sensing into a single system, expanding the utility of porous materials across energy, environmental, and biomedical domains.

- Stimuli-Responsive and Adaptive Materials

Dynamic, stimuli-responsive porous systems represent a major frontier. Materials that adapt their structure, surface chemistry, or functionality in response to environmental cues—such as pH, temperature, light, ionic strength, current voltage, or redox potential—can enable unprecedented performance in smart filtration, on-demand drug release, adaptive catalysis, and environmental remediation. Future research will likely explore hybrid materials that combine inorganic frameworks with responsive polymers or bioactive ligands, achieving autonomous modulation of adsorption, catalytic activity, or molecular release. These adaptive systems will allow materials to respond to complex, time-varying environments in real-world applications.

- Bridging Laboratory and Field Applications

To translate promising laboratory results into practical technologies, future studies must evaluate porous materials under realistic operational conditions. This includes industrial effluents, complex aqueous matrices, atmospheric pollutants, and biologically relevant fluids. Long-term stability, fouling resistance, regeneration capability, and performance under fluctuating environmental conditions will be critical metrics. Research that integrates device-level testing—such as in membrane modules, catalytic reactors, or implantable scaffolds—will bridge the gap between materials science and engineering, ensuring functional performance in real-world applications.

- Hybrid and Multifunctional Systems

The next generation of porous materials will increasingly exploit hybridization across material classes. For instance, combining MOFs with polymers, porous metal oxides with metallic nanoparticles, or biochars with functional ligands can create synergies that exceed the performance limits of individual components. Such hybrid systems are poised to deliver multi-modal functionality—for example, simultaneous adsorption and catalysis, dual drug delivery and imaging, or combined CO_2_ capture and pollutant degradation—enabling broader applicability across energy, environment, and biomedical sectors.

- Mechanistic Understanding and Predictive Science

Fundamental insight into adsorption kinetics, catalytic mechanisms, mechanical behavior, and degradation pathways will remain central. Future research will leverage advanced characterization techniques (in situ spectroscopy, electron microscopy, tomography) and multiscale modeling to elucidate how molecular-scale interactions govern macroscopic performance. Such mechanistic understanding will allow rational design of materials that are not only highly effective but also predictable, reproducible, and scalable.

- Interdisciplinary Collaboration and Societal Integration

Finally, the evolution of nanoporous materials science will depend on interdisciplinary collaboration. Chemists, materials scientists, engineers, environmental scientists, and biomedical researchers must work together to integrate synthesis, modeling, device design, and real-world testing. Close engagement with industry, policymakers, and regulatory bodies will ensure that these materials address societal needs effectively, from air and water purification to energy storage, climate mitigation, and advanced therapeutics.

In summary, the future of nanoporous and mesoporous materials is characterized by convergence: of sustainability with high performance, of computational design with experimental validation, of hierarchical structures with stimuli-responsive functionality, and of fundamental understanding with real-world implementation. By embracing these directions, researchers will be able to create materials that not only meet technical specifications but also address pressing environmental, energy, and health challenges on a global scale.
